# Supplementary material for: Effects of Perturbation Velocity, Direction, Background Muscle Activation, and Task Instruction on Long-Latency Responses Measured From Forearm Muscles
Source: Front Hum Neurosci. 2021 Apr 16;15:639773. doi: 10.3389/fnhum.2021.639773 (PMC8085277; doi:10.3389/fnhum.2021.639773)
Supplement: Supplementary file 1 [file Data_Sheet_1.pdf]

Supplementary materials

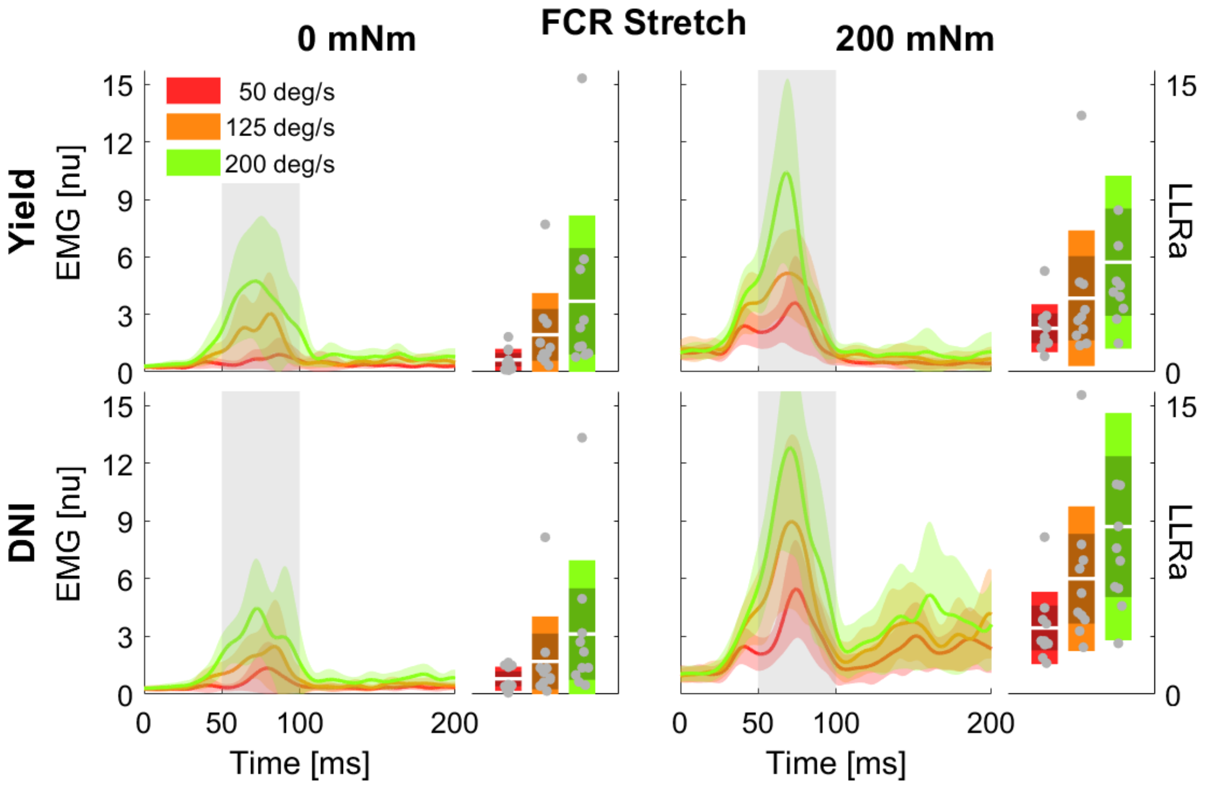

Supplementary Figure 1. Averaged EMG time series from all subjects for the stretched state of the FCR. Values are in normalized units. Line color or patch color represents the speed of the perturbation (red = 50 deg/s, orange = 125 deg/s, green = 200 deg/s). Plots split by row are split by instruction, and plots split by column are split by background torque. Distributions to the right of the time series are the mean EMG within the LLR (shaded region: 50-100 ms) for the three velocities. Darker shades represent 1 standard deviation from the mean, and lighter shades indicate the 95% confidence interval. Subject averages are plotted on top of the bars.

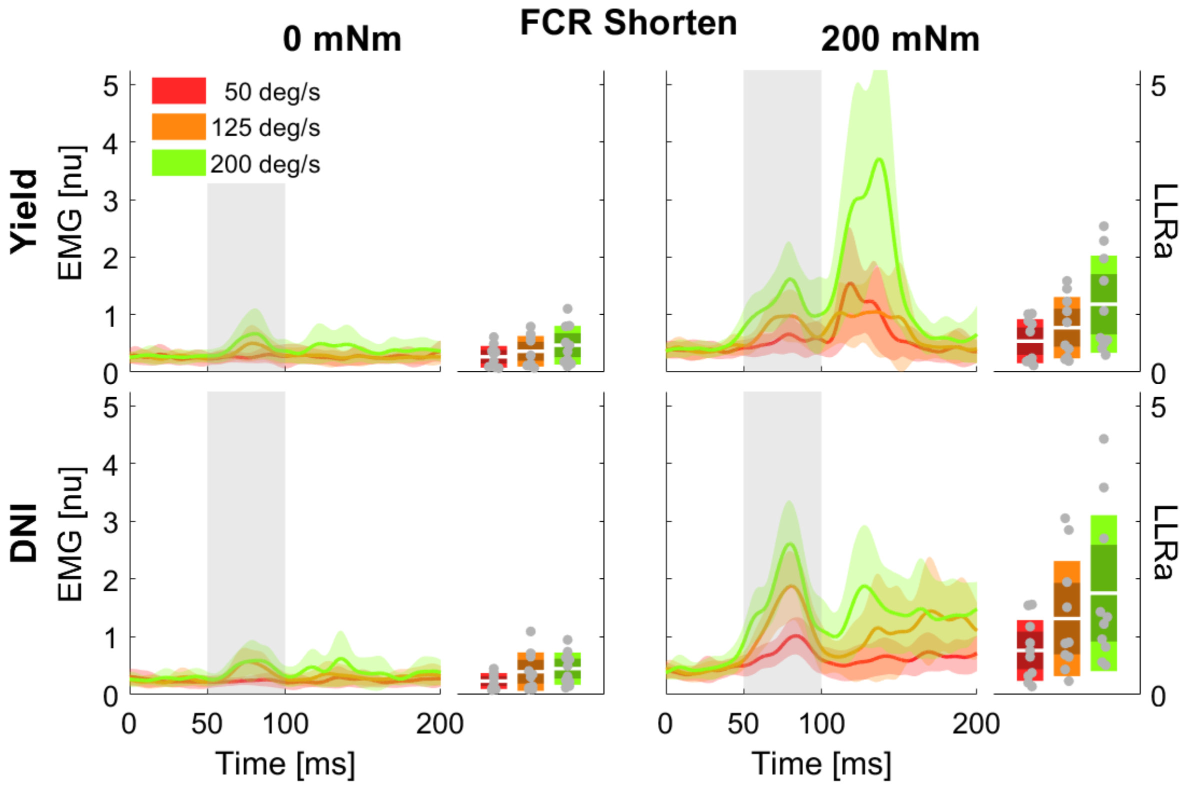

Supplementary Figure 2. Averaged EMG time series from all subjects for the shortened state of the FCR. Values are in normalized units. Line color or patch color represents the speed of the perturbation (red = 50 deg/s, orange = 125 deg/s, green = 200 deg/s). Plots split by row are split by instruction, and plots split by column are split by background torque. Distributions to the right of the time series are the mean EMG within the LLR (shaded region: 50-100 ms) for the three velocities. Darker shades represent 1 standard deviation from the mean, and lighter shades indicate the 95% confidence interval. Subject averages are plotted on top of the bars.
